# COVID-19 lockdown effects on the foraging strategies of a facultative scavenger

**DOI:** 10.1098/rsbl.2025.0223

**Published:** 2025-10-29

**Authors:** Benedetta Catitti, Ying-Chi Chan, Damien R. Farine, Steffen Oppel, Florian Orgeret, Patrick Scherler, Matthias Tschumi, Stephanie Witczak, Martin U. Grüebler

**Affiliations:** ^1^ Swiss Ornithological Institute, Sempach, Switzerland; ^2^ Nanyang Technological University, Singapore, Singapore; ^3^ Department of Evolutionary Biology and Environmental Studies, University of Zurich, Zurich, Switzerland; ^4^ Division of Ecology and Evolution, Research School of Biology, Australian National University, Canberra, Australian Capital Territory, Australia; ^5^ Department of Collective Behavior, Max Planck Institute of Animal Behavior, Radolfzell, Germany; ^6^ La Rochelle Université, La Rochelle, France

**Keywords:** pandemic, anthropogenic food, roadkill, human–wildlife interactions, red kite, urban ecology, step-selection functions

## Abstract

Human activity has profoundly shaped the landscape of resources available to animals. While certain species, such as scavengers, are particularly adapted to exploit resources that fluctuate significantly over space and time, their responses to sudden human-induced changes in resources remain poorly understood. The COVID-19 lockdown offered a natural experiment to study these dynamics, as reduced human mobility abruptly decreased roadkill availability for scavengers. Here, we examined how reductions in roadkill affected the foraging behaviour of an avian facultative scavenger, the red kite (*Milvus milvus*). We hypothesized that with fewer carcasses available, red kites would decrease their use of roads for scavenging. GPS tracking data from 199 non-breeding individuals confirmed that red kites switched from actively selecting roads before the lockdown (2017–2019) to avoiding them during lockdown (2020), with the trend reversing again afterwards (2021–2023). Selection for areas with higher probability of anthropogenic feeding increased during lockdown and remained elevated afterwards. Our findings highlight that abrupt changes in human activity can drive rapid behavioural shifts in a generalist forager, with certain effects lasting for years after the change.

## 1. Introduction

Human activity can influence both the spatiotemporal distribution and the abundance of food resources for animals, leaving them susceptible to sudden changes in human behaviour [1–3]. At the individual level, human-driven changes in resource distribution and abundance may drive compensatory behavioural adjustments. For example, a sudden decline in key anthropogenic food sources may cause individuals to exploit less-preferred resources or increase foraging effort [4]. At the population level, changes in human activity can lead to the spatial redistribution of individuals and affect the overall competition for the available resources [5]. However, understanding the causal drivers of these patterns is challenging, as we are limited in our ability to experimentally manipulate systems to drive population-level changes.

Human-driven behavioural changes of animals may also affect human health. Scavengers provide vital ecosystem services by controlling disease and recycling nutrients [6–8], by feeding on roadkill [9], food scraps at dumpsites [10], discards from fisheries [11] and carcasses in urban environments [12]. Scavengers also exploit a variety of human-provisioned food sources, such as in private gardens [1,2,13]. Anthropogenic resources can shape local densities of scavengers [14] and make them particularly sensitive to changes in human behaviour, with consequences for survival [15,16]. While research on foragers dependent on anthropogenic food sources—including scavengers—has focused on how they adapt to changes in food distribution [5,17] and the fitness consequences of increased reliance on anthropogenic foods (e.g. [18], reviewed in [19]), the effects of sudden fluctuations in food abundance on their foraging behaviour have received less attention (but see [4]).

In 2020, the global COVID-19 pandemic led to widespread restrictions on human mobility in many countries, as governments implemented travel bans to reduce the spread of the virus. These so-called ‘lockdowns’ provided a unique natural experiment to examine how animals respond to abrupt alterations in human activity. During this period, many animals that typically relied on anthropogenic food sources—either directly provisioned or indirectly available through human activities—were reported to change their diets [20,21]. The reduction in human mobility led to massive decreases in traffic volume and substantially reduced the amount of roadkill, an opportunistic resource for scavengers (e.g. [22,23]). By reshaping the resource landscape, changes in human mobility represented a natural experiment allowing us to determine whether human-driven food availability influences the behaviour of scavengers. Specifically, we could test whether scavengers shifted their habitat preference from roads to other feeding sites and whether these changes persisted post-lockdown.

Here, we quantify whether habitat selection by a facultative scavenger, the red kite (*Milvus milvus*), changed during the COVID-19 lockdown in 2020. Red kites represent an excellent system to investigate the effects of changes in food availability on foraging behaviour because they exploit a mixture of food sources, including actively hunted small mammals, roadkill carrion and anthropogenic food scraps [24]. Using integrated step-selection functions, we analysed 386 annual GPS tracks from 199 non-breeding red kites in Switzerland to assess how selection for scavenging habitats—roads and areas with anthropogenic food—changed during the COVID-19 lockdown (March–May 2020) compared to pre-lockdown (2017−2019) and post-lockdown (2021−2023) periods, while accounting for natural prey availability. The post-lockdown data enable us to assess whether lockdown-induced behavioural changes had long-term effects. Assuming that scavenging opportunities influence red kite movement decisions, we predict a decline in selection for roads during lockdown relative to the pre- and post-lockdown periods and an increase in selection for alternative sources of anthropogenic food.

## 2. Methods

### (a) Study species and study area

Our study focuses on a population of red kites in western Switzerland, in an area extending 387 km^2^ (see [25] for details on the study area). Red kites are birds of prey that hunt small rodents and scavenge on carcasses and food scraps [26]. Previous surveys have estimated that up to 0.9 kg of anthropogenic food per km^2^ is available to the kites in the study area daily, including both intentional (20%) and unintentional food provisioning (80%), but excluding roadkill [27]. The study area reflects a typical Swiss lowland landscape characterized by extensive human infrastructure, with one of the highest railway and motorway densities in Europe [28,29]. This dense human population and road density contribute to widespread wildlife–traffic collisions [30].

### (b) GPS tagging

Between 2017 and 2022, we equipped red kites with solar-powered GPS-GSM-UHF transmitters (Ecotone SKUA/CREX and Milsar M9) using backpack-style diagonal-loop harnesses (see [31] for details on tagging procedures). GPS loggers were set to record locations every hour (Ecotone) or every 10 min (Milsar) between 03.00 and 21.00 h UTC (05.00 to 23.00 h local time in summer and 04.00 to 22.00 h local time in winter).

### (c) Telemetry data

**Figure 1.**
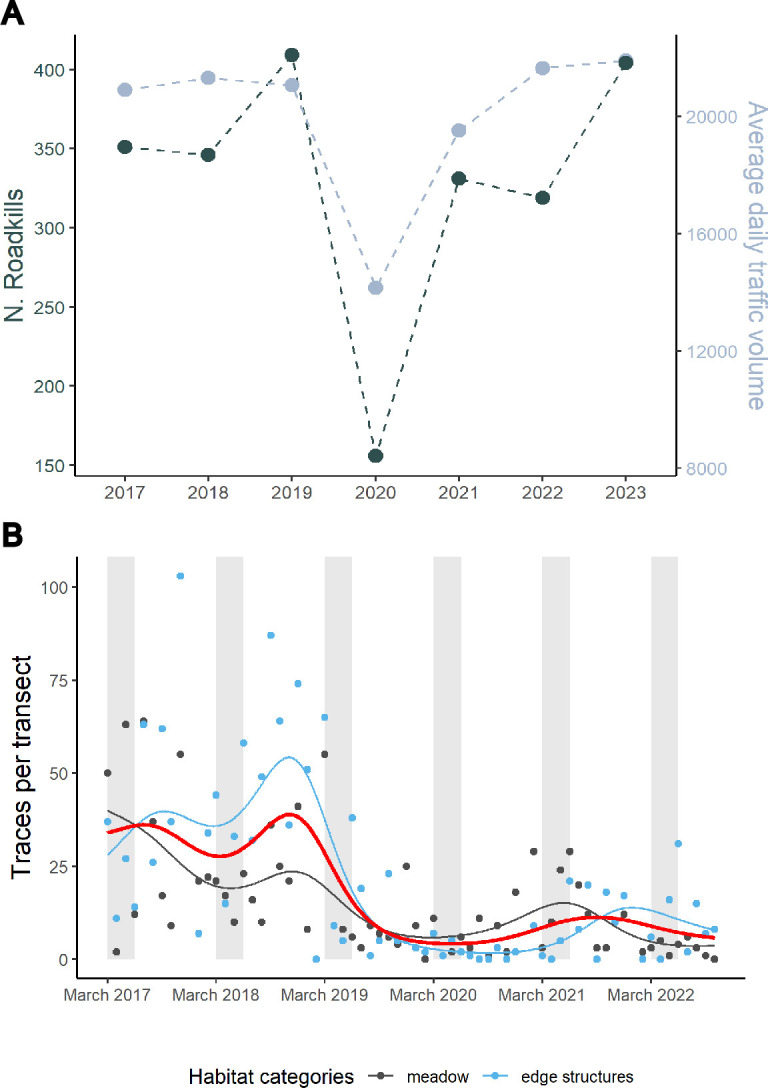
Key environmental changes affecting red kite food availability during the study period. (A) Roadkill collected by game keepers (dark blue, left *y*-axis) and average daily traffic volume (ADT, light blue, right *y*-axis) in the Canton of Fribourg (Switzerland) between March and May 2017−2023. Traffic data were collected on four main roads (two motorways, two primary roads) from the Federal Roads Office monthly summaries, then averaged annually. (B) Rodent availability from monthly rodent surveys across habitats; grey bands highlight study periods (March–May). Thin lines show model predictions for meadows (black) and edge structures (blue), and the red line represents the average predicted rodent index between habitats. Points are raw data.

We analysed GPS data collected during the main national COVID-19 lockdown in Switzerland (15 March–15 May 2020) [32,33] and from the same period in the three preceding (2017−2019) and the three following years (2021−2023). Data from the canton of Fribourg—where most of our study area was situated—showed that both traffic volume and roadkill decreased considerably during lockdown compared to the non-lockdown years (figure 1).

Given that the lockdown period overlapped with the breeding season, during which breeding birds have a restricted home range, we only used data from non-breeders whose space use is not confounded by parental duties at a nest. From these data, we first removed movement outliers (>35 m s^–1^ for 1-h intervals, [34]; <0.001% of data), and retained only daytime points (07.00–19.00 h CEST) because red kites are diurnal foragers. We also excluded periods of likely active migration (3% of the points; see electronic supplementary material, S1 for details). After filtering, we resampled tracks at 1-h intervals—the lowest common sampling frequency across individuals.

### (d) Integrated step-selection analysis

#### (i) Individual step calculations

For each individual, we extracted hourly ‘steps’ (i.e. movement between two consecutive hourly GPS fixes) and discarded steps with longer durations (e.g. overnight or during times of temporary data sparsity). We then created 15 random steps for each observed step using the ‘amt’ package [35] to allow us to contrast the habitat the bird actually used (observed) with potentially available (random) habitat at that step. Random steps were generated by using step lengths drawn from an exponential distribution based on the observed step lengths across the population [36] and turning angles from a uniform distribution spanning from –π to π. After filtering out locations for which no covariate data were available (see below), our final dataset consisted of 95 702 observed and 1 435 530 random steps from 199 non-breeding individuals across 7 years (N bird-seasons = 386; N_2017_ = 50, N_2018_ = 90, N_2019_ = 89, N_2020_ = 60, N_2021_ = 45, N_2022_ = 49, N_2023_ = 3).

#### (ii) Covariates

#### Distance to road

From the OpenStreetMap road database (https://download.geofabrik.de), we selected all major paved roads (motorways and cantonal roads connecting large towns; see electronic supplementary material, figure S1) and railroads, hereafter collectively referred to as ‘roads’. Using this dataset, we generated a 30 m resolution raster where each cell value represented the distance to the nearest road. At the end location of each step, we then extracted the corresponding distance to the nearest road.

#### Anthropogenic feeding

We used predictions from a previous study that combined surveys at households with red kite tracking data to predict the spatially explicit probability of anthropogenic feeding at a 500 × 500 m resolution across Switzerland [37]. For each step, we extracted the corresponding probability of anthropogenic feeding as the value of the 500 m cell where this step ended. The anthropogenic feeding probability could only be predicted in areas with sufficient red kite tracking data and we thus excluded data from all other areas.

#### Natural food availability (rodent index)

To estimate natural red kite prey abundance, we conducted monthly trace surveys [38], from 2017 to 2022, for water voles (*Arvicola terrestris*) and common voles (*Microtus arvalis*) within our study area and estimated a daily index for herbivorous rodent abundance. For 2023, the year where no rodent data were collected and with the lowest GPS data contribution, we averaged the rodent index values from the two preceding years (see electronic supplementary material, S3 for details). While these counts do not cover the full extent of the red kites’ movements in Switzerland, a significant proportion of tracking data (69%) was within the area of rodent sampling (electronic supplementary material, figure S2).

#### Age

All birds were tagged as fledglings in June–July. Consequently, the lockdown period (March–May) corresponded, at the earliest, to their second calendar year (age 2). Second and third calendar year birds were the most represented age class (N_age2_ = 134, N_age3_ = 146, N_age4_ = 62, N_age5_ = 26, N_age6_ = 15, N_age7_ = 3). To test whether experience influenced the response to changes in roadkill, we classified individuals aged 2 as ‘young’ and all older individuals as ‘old’.

None of the variables described above were correlated with each other, except for the rodent index and the lockdown (0/1) and post-lockdown (0/1) covariates (electronic supplementary material, figure S3). In the lockdown year of 2020, the rodent index was substantially lower than in the pre-lockdown years (Pearson’s *r* = −0.65 and −0.47, *p <* 0.001 electronic supplementary material, figure S3). This decline was not due to data collection limitations or imposed changes in farming practice but reflected a natural reduction in rodent abundance that coincided with the COVID-19 lockdown. All numeric variables were standardized using a z-score transformation and back-transformed for plotting.

### (e) Integrated step-selection analysis

We compared observed versus random steps using a Poisson regression model implemented in the ‘glmmTMB’ package [39], following the approach described by Muff *et al*. [40]. This method allows the inclusion of individual random slopes and requires fixing the variance of the stratum-specific intercept to an arbitrary high value (here 10^6^). The model included two movement parameters, namely the cosine of the turning angle and the natural logarithm of step length, which we interacted with a binary variable time of day because step lengths were shorter in the morning (before 10.00: level 0) and evening (after 5.00: level 0) compared to midday (between 10.00 and 5.00: level 1; electronic supplementary material, figure S4), likely due to the reduced availability of thermals during cooler parts of the day. To test our main hypothesis—whether red kites selected fewer roads during lockdown—we examined the interaction between distance to road and lockdown status (2020 versus 2017−2019 and versus 2021−2023), including a quadratic term for distance to road (table 1). This quadratic term accounted for the possibility that selection occurs at intermediate distances, because birds may avoid roads, and also areas very far from roads, because these areas are unlikely habitat for red kites (e.g. in mountains or forests). In addition, we included the interaction of rodent index with distance to road to allow that the attraction of roads may vary with natural food availability. We accounted for differences in selection strength due to experience by including age as a variable and an interaction between age and distance to road, given that age-related foraging experience may shape the attraction of roads. We also tested whether red kites switched to alternative anthropogenic food sources during lockdown by including the probability of anthropogenic feeding at a given step as a covariate interacting with lockdown status. Anthropogenic feeding also interacted with age and rodent index following the same rationale as for the interactions with distance to road. To account for individual variability in road and feeding site selection, we included random slopes for distance to road and anthropogenic feeding for each bird-year combination (table 1). To test other plausible interactions between habitat and movement covariates we ran a forward model selection based on Akaike’s information criterion [41] (see electronic supplementary material, S6). The final model’s predictive power was evaluated by k-fold cross-validation following Fortin *et al.* [42], which correlated the frequency of a predicted step with the rank of its predicted nature (see electronic supplementary material, S7 for details).

**Table 1.** Covariates included in an integrated step-selection function (iSSF) examining the effect of COVID-19 lockdown phases on habitat selection of non-breeding red kites in Switzerland between 2017 and 2023, with ecological justifications for each variable. The base model accounts for movement parameters (step length and turning angle), while additional terms test hypotheses about attraction for roads and anthropogenic feeding sites, natural food availability (rodent index), time of day and age class. Random effects account for individual variation (*id* = individual-year identifier).

	terms	predictions
base model	ln(step length) + cos(turning angle)	individuals have movement preferences towards certain step lengths and turning angles. The step length is higher during midday hours, it can differ according to the period (lockdown versus non-lockdown), the distance to the closest road and the probability of anthropogenic feeding in a given location.
	ln(step length) × time of day	
	ln(step length) × lockdown + ln(step length) × post-lockdown	
	ln(step length) × distance to road	
	ln(step length) × distance to road^2^	
	ln(step length) × anthropogenic feeding	
road attraction	distance to road + distance to road^2^	selection for short distances to road is weaker during COVID-19 lockdown due to low human mobility reducing roadkill availability
	distance to road × lockdown(0/1)	
	distance to road × post-lockdown(0/1)	
	distance to road^2^ × lockdown(0/1)	
	distance to road^2^ × post-lockdown(0/1)	
	distance to road × rodent index	selection for short distances to road is stronger when natural food availability is low
	distance to road^2^ × rodent index	
	distance to road × time of day	selection for short distances to road is stronger in midday hours
	distance to road^2^ × time of day	
	distance to road × age(young/old)	selection for short distances to road is stronger in younger individuals
	distance to road^2^ × age(young/old)	
attraction to areas of high anthropogenic feeding probability	anthropogenic feeding	selection for areas of high anthropogenic feeding probability is stronger during COVID-19 lockdown
	anthropogenic feeding × lockdown(0/1)	
	anthropogenic feeding × post-lockdown(0/1)	
	anthropogenic feeding × rodent index	selection for areas of high anthropogenic feeding probability is stronger when natural food availability is low
	anthropogenic feeding × age(young/old)	selection for areas of high anthropogenic feeding probability is stronger in younger individuals
random structure	distance to road|id	account for individual-level variation in selection for distances to roads and areas of varying anthropogenic feeding probability
	anthropogenic feeding|id	

### (f) Results

The k-fold cross-validation results indicated that the final model performed significantly better than random chance (mean correlation coefficient between frequency and rank −0.915, 95% CI: −0.922 to −0.906; electronic supplementary material, figure S5). The movement behaviour of non-breeding red kites was influenced by habitat covariates and differed between the three study periods (table 2).

**Table 2.** Estimates from an integrated step-selection model examining the habitat selection of non-breeding red kites in Switzerland between 2017 and 2023. The model contains (i) movement covariates, describing animal movement preference, (ii) habitat covariates, describing habitat preferences and (iii) interactions between the two (M × H). All continuous covariates were extracted at the end of each step. Model estimates with 95% confidence intervals (95% CI) not overlapping 0 are reported in bold. Random effects account for individual variation (*id* = individual-year identifier).

	terms	estimate	95% CI	
movement	ln(step length)	**−0.265**	**−0.277**	**−0.253**
	cos(turning angle)	**−0.430**	**−0.437**	**−0.424**
	ln(step length) × time of day[midday]	**0.561**	**0.547**	**0.576**
	ln(step length) × lockdown	0.018	−0.001	0.038
	ln(step length) × post-lockdown	**0.033**	**0.015**	**0.050**
habitat	distance to road	−0.056	−0.165	0.052
	distance to road^2^	**−0.296**	**−0.323**	**−0.269**
	distance to road × lockdown [1]	**0.486**	**0.252**	**0.719**
	distance to road^2^ × lockdown [1]	**−0.103**	**−0.164**	**−0.043**
	distance to road × post-lockdown [1]	0.143	−0.052	0.338
	distance to road^2^ × post-lockdown [1]	0.002	−0.047	0.051
	distance to road × rodent index	**0.204**	**0.120**	**0.287**
	distance to road^2^ × rodent index	**−0.033**	**−0.060**	**−0.005**
	distance to road × time of day[midday]	**−0.046**	**−0.077**	**−0.016**
	distance to road^2^ × time of day[midday]	**0.072**	**0.054**	**0.089**
	distance to road × age[young]	−0.111	−0.235	0.013
	distance to road^2^ × age[young]	−0.001	−0.027	0.024
	anthropogenic feeding	**0.069**	**0.026**	**0.093**
	anthropogenic feeding × lockdown [1]	**0.089**	**0.007**	**0.171**
	anthropogenic feeding × post-lockdown [1]	**0.094**	**0.027**	**0.161**
	anthropogenic feeding × rodent index	−0.003	−0.038	0.031
	anthropogenic feeding × age[young]	0.006	−0.030	0.043
MxH	ln(step length) × distance to road	**0.040**	**0.031**	**0.050**
	ln(step length) × distance to road^2^	**0.029**	**0.022**	**0.037**
	ln(step length) × anthropogenic feeding	**0.035**	**0.029**	**0.042**
	*random effects*			
	*distance to road|id*	0.485	0.442	0.532
	*anthropogenic feeding|id*	0.110	0.097	0.125

Shorter step lengths, indicative of slower movement, were associated with proximity to roads and low anthropogenic feeding probability. Additionally, time of day strongly affected step length, with longer steps occurring during mid-day compared to morning and evening hours (table 2).

**Figure 2.**
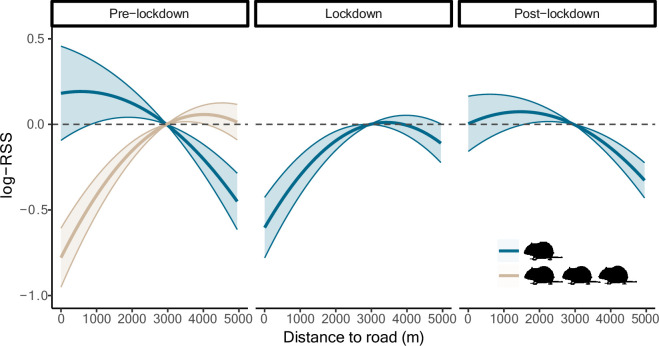
Effect of distance to roads on red kite relative selection strength (log-RSS) from March to May during pre-lockdown (2017−2019), lockdown (2020) and post-lockdown (2021−2023). Positive log-RSS values indicate selection for a particular location, negative values indicate avoidance. Shaded areas show 95% CI. The *x*-axis is limited to a representative range of distances (<5000 m; 82% of locations). Effects are predicted under low (blue) and high (beige) rodent index values, the latter shown only for pre-lockdown, as sufficient variation in rodent activity was only present then.

Red kites exhibited positive selection for areas near roads during the pre-lockdown period, avoidance during lockdown and no selection post-lockdown (table 2; figure 2). Selection for roads increased when natural food availability (rodent index) was low and, to a lesser extent, during midday. Young birds showed stronger selection for roads than older birds, but the 95% CI of this effect included 0 (table 2).

**Figure 3.**
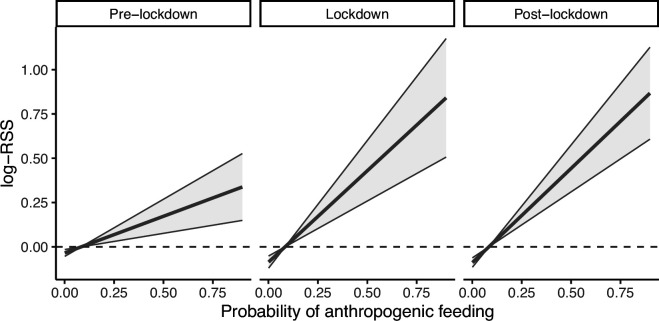
Effect of anthropogenic feeding probability on red kite relative selection strength (log-RSS) from March to May during pre-lockdown (2017−2019), lockdown (2020) and post-lockdown (2021−2023). Positive log-RSS values indicate selection for a particular location, negative values indicate avoidance. Shaded areas show 95% CI.

Red kites showed selection for areas with high probability of anthropogenic feeding across all study periods, but selection was significantly higher during lockdown and post-lockdown than during pre-lockdown (table 2; figure 3). This pattern was slightly stronger in the morning and evening hours and was not influenced by rodent availability or age. Individuals responded more homogeneously to anthropogenic feeding compared to roads (electronic supplementary material, figure S6).

## 3. Discussion

The response to the global COVID-19 pandemic provided a unique opportunity to study the large-scale impacts of human activity on animal behaviour [43,44]. While many studies documented behavioural changes during this period, uncovering the underlying mechanisms has proven challenging. Research focusing on foraging behaviour [45,46] suggested that the direction and magnitude of effects of altered human mobility depend on a species' reliance on anthropogenic food sources [44,45]. Our results show that red kites significantly adjusted their foraging strategies in response to changes in human-driven food availability during lockdown. Responding to decreased human mobility and consequent scarcity of roadkill during lockdown, red kites showed a marked shift in their attraction to roads, with birds favouring vicinity to roads before and after lockdown but avoiding roads during lockdown. The avoidance of roads during lockdown was even more striking because it coincided with low abundance of natural prey, a condition where greater attraction to roadkill would be expected. Furthermore, birds increased their selection for areas with a high probability of anthropogenic feeding during lockdown, a pattern that persisted afterwards. These findings highlight the behavioural flexibility of red kites in adapting to changes in the food landscape. This flexibility aligns with classical ecological niche theory, which predicts generalist species to be well equipped to adjust to rapid environmental changes [47–50].

Our results showing changes in selection for roads and areas of anthropogenic feeding suggest that the red kite’s response to human-induced changes in food availability is part of a broader behavioural flexibility shaped by natural food fluctuations. Prior to lockdown, selection for locations near roads was strongest when natural food was scarce, highlighting the opportunistic nature of scavenging in this species [51]. When both roadkill and natural prey declined during the lockdown, red kites increasingly used areas of high probability of anthropogenic feeding, a preference that remained elevated under low natural prey conditions, even after human mobility restrictions were lifted. This lasting shift in selection for anthropogenic feeding sites could potentially be because of the discovery of reliable new food sources, which they continued to exploit due to high accessibility. Additionally, anthropogenic food might help compensate for the low levels of natural prey [19,52]. Since our study focuses on immature individuals, foraging behaviours developed during lockdown might be formative, potentially shaping long-term habits of anthropogenic food reliance and spreading through social learning in the following years. Having the flexibility to modify the foraging behaviour to maximize a wide range of natural and anthropogenic food sources is likely an important factor contributing to the demographic recovery of this species across its distributional range [13,53,54].

The behavioural adjustments observed in this study may have implications for the individuals beyond food acquisition. Although roadkill offers an easily accessible food source for scavengers [9], it also carries significant risks, particularly from vehicle collisions [55]. Therefore, the reduced use of roads during the lockdown may improve survival, though population-level impacts require assessing how decreased human mobility affected breeding birds as well. The behavioural flexibility exhibited by red kites may also generate cascading ecological effects such as alleviating interspecific food competition and predation pressure on natural prey [56,57]. The increasing reliance of red kites on anthropogenic food sources—whether driven by natural food scarcity or reduced availability of roadkill—may also increase human–wildlife conflict. While some people intentionally feed raptors, large scavengers, like the red kite, are often met with ambivalence or opposition from local communities [58]. Their increased presence at human-provided food sources during prey shortages can heighten negative perceptions—especially in urban areas, where high human density intensifies wildlife–human tensions [59]. Furthermore, an increased use of anthropogenic feeding sites could raise disease transmission risks, as contact rates increase and pathogens accumulate in large gatherings of individuals at feeding locations [60,61].

One confounding factor of our study was the low rodent abundance in 2020, which coincided with the reduced human mobility during COVID-19 lockdowns. While we cannot exclude that natural prey fluctuations had an effect on selection patterns, red kites responded contrary to expectations. During times of low natural prey, facultative scavengers are generally expected to increase foraging around anthropogenic food sources, including roads where roadkill may be found. Instead, we observed reduced selection for roads. This response strengthens our conclusion that reduced human mobility, not natural food availability, drove changes in foraging behaviour, likely by reducing the roadkill available to scavengers. The increased use of anthropogenic feeding sites during and after the COVID-19 lockdown may, however, reflect both reduced roadkill availability and lower natural food abundance. A limitation of our study is the lack of data on anthropogenic feeding trends across years. If anthropogenic feeding increased during COVID-19 lockdowns, as observed elsewhere [62], it could have helped offset the decline in food availability from roads and railways, potentially reinforcing the use of anthropogenic feeding sites by non-breeding kites during and after lockdown. Future research should integrate human and wildlife behaviours at matched temporal scales to clarify these ecological relationships.

Altogether, this study shows that changes in human mobility due to COVID-19 restrictions reduced the foraging behaviour of a generalist predator in areas that typically have high rates of roadkill, and we encourage researchers to test whether human behaviour affects other species similarly in different human-modified landscapes [63]. Anthropized environments can generate ecological traps, where adaptations may have hidden costs [64]. Further research is needed to assess how widespread behavioural adaptations are across species and human-modified regions, and whether such behavioural shifts ultimately enhance or reduce long-term fitness of individuals.

## Data Availability

Data and R code are available from the Zenodo Repository [65]. Supplementary material is available online [66].
